# Hypoxia-Induced Down-Regulation of microRNA-34a Promotes EMT by Targeting the Notch Signaling Pathway in Tubular Epithelial Cells

**DOI:** 10.1371/journal.pone.0030771

**Published:** 2012-02-17

**Authors:** Rui Du, Wenjuan Sun, Lin Xia, Ali Zhao, Yan Yu, Lijuan Zhao, Hanmin Wang, Chen Huang, Shiren Sun

**Affiliations:** 1 Department of Nephrology, Xijing Hospital, Fourth Military Medical University, Xi'an, People's Republic China; 2 State Key Laboratory of Cancer Biology & Xijing Digestive Hospital, Fourth Military Medical University, Xi'an, People's Republic China; Istituto Dermopatico dell'Immacolata-IRCCS, Italy

## Abstract

**Background:**

Hypoxia-induced renal tubular cell epithelial–mesenchymal transition (EMT) is an important event leading to renal fibrosis. MicroRNAs (miRNAs) are small non-coding RNA molecules that bind to their mRNA targets, thereby leading to translational repression. The role of miRNA in hypoxia-induced EMT is largely unknown.

**Methodology/Principal Findings:**

miRNA profiling was performed for the identification of differentially expressed miRNAs in HK-2 cells under normal and low oxygen, and the results were then verified by quantitative real time RT-PCR (qRT-PCR). The function of miRNAs in hypoxia-induced renal tubular cell EMT was assessed by the transfection of specific miRNA inhibitors and mimics. Luciferase reporter gene assays and western blot analysis were performed to validate the target genes of miR-34a. siRNA against Jagged1 was designed to investigate the role of the miR-34a-Notch pathway in hypoxia induced renal tubular cell EMT. miRNA-34a was identified as being downregulated in hypoxic renal tubular epithelial cells. Inhibition of miR-34a expression in HK-2 cells, which highly express endogenous miR-34a, promoted a mesenchymal phenotype accompanied by reduced expression of the epithelial marker Z0-1, E-cadherin and increased expression of the mesenchymal markers α-SMA and vimentin. Conversely, miR-34a mimics effectively prevented hypoxia-induced EMT. Transfection of miRNA-34a in HK-2 cells under hypoxia abolished hypoxia-induced expression of Notch1 and Jagged1 as well as Notch downstream signals, such as snail. Western blot analysis and luciferase reporter gene assays showed direct evidence for miR-34a targeting Notch1 and Jagged1. siRNAs against Jagged1 or Notch1 effectively prevented miR-34a inhibitor-induced tubular epithelial cell EMT.

**Conclusions/Significance:**

Our study provides evidence that the hypoxia-induced decrease of miR-34a expression could promote EMT in renal tubular epithelial cells by directly targeting Notch1 and Jagged1, and subsequently, Notch downstream signaling.

## Introduction

MicroRNAs (miRNAs) are a class of non-coding, single-stranded, small RNA molecules about 19–25 nucleotides in length, which negatively regulate gene expression at the post-transcriptional level through nucleotide base pairing between complementary sequences of miRNAs and 3′-untranslated regions (3′UTR) of messenger RNAs (mRNAs) [1]. It has been suggested that miRNAs are involved in embryonic development, tumorigenesis, metastasis, metabolism, and many other physiological and pathological processes [2]. The biological functions of most miRNAs are not yet fully understood. Recently, miRNAs were demonstrated to be involved in the process of epithelial–mesenchymal transition (EMT) by modulation of EMT-related genes. EMT is characterized by the loss of cell polarity and epithelial surface markers, induction of the expression of mesenchymal markers, and increased motility and invasiveness [3]. Several studies have shown that members of the miR-200 family (e.g., miR-141 and miR-200b) and miR-205 can prevent transforming growth factor β (TGF-β) induced EMT by downregulating ZEB1 and ZEB2, the two major transcriptional repressors of E-cadherin, which is a key marker of epithelial cells [4]–[6]. miR-192 was also found to repress the E-Box repressors ZEB1 and ZEB2 in tubular epithelial cells and increase collagen 1-α2 production in mesangial cells [7], [8]. In human renal biopsies, low expression of miR-192 correlated with tubulointerstitial fibrosis and low estimated GFR [8]. These data suggested that some miRNA species may play important roles in tubular epithelial cell EMT and renal fibrosis.

Chronic hypoxia is one of the final pathways that lead to end stage renal failure [9]. Recently, it has been well established that activation of HIF-1 signaling in renal epithelial cells under low oxygen is associated with the development of chronic renal disease and may promote fibrogenesis [10], [11]. HIF-1 binds to the promoters of a wide range of target genes through hypoxia-responsive element and trans-activates certain EMT regulators, such as Snail, Zeb1, SIP1, E47/TCF3, CTGF, and LOX (lysyl oxidase) [12]–[15]. We previously reported that the activation of Twist and URG11 due to hypoxia in renal epithelial cells plays an important role in renal fibrosis and the progression of EMT in renal epithelial cells [16], [17]. Considering that HIF-1 is strongly regulated by hypoxia and could trans-activate a wide variety of transcripts through hypoxia-responsive elements (HREs) in the promoters of target genes, it is not surprising that HIF-1 could regulate miRNA transcripts by binding to the promoters of target miRNAs. In fact, increasing evidences have shown that hypoxia-regulated miRNAs (HRMs) exhibit induction in response to HIF activation and participate in the development of tumorigenesis and angiogenesis [18], [19], although there are no data regarding the role of miRNAs in hypoxia-induced EMT and renal fibrosis.

The Notch signaling pathway is an evolutionarily conserved pathway that regulates development by controlling cell fate determination, cell proliferation, differentiation, and apoptosis during embryonic and postnatal stages [20].In mammals, the Notch family consists of 4 transmembrane receptors (Notch1–Notch4) and 5 ligands (Jagged-1, Jagged-2, Delta-1, Delta-3, and Delta-4). All of the receptors have an extracellular domain containing multiple epidermal growth factor (EGF; 131530)-like repeats and an intracellular region containing the RAM domain, ankyrin repeats, and a C-terminal PEST domain [21]. After Notch receptors are triggered by the binding of the Notch ligands, the Notch intracellular domain (NIC) is cleaved by a proteinase complex containing γ-secretase. NIC is released from the plasma membrane and translocates into the nucleus, where it forms a complex with RBP-Jk/CBF1, Su(H), Lag-2 (RBP-Jk/CSL), and mastermind-like (MAML) [22], [23]. This protein complex will recruit other transcription co-activators and transactivate the transcription of target genes, such as the Hes family basic helix-loop-helix members and others [24]. Increased Notch activity due to enhanced Jagged-1 ligand and Notch1 expression has also been detected in fibrotic kidneys and therefore may be relevant to the pathogenesis of renal fibrosis [25], [26]. Moreover, hypoxic induction of EMT in renal epithelial cells may also involve HIF-mediated transcriptional upregulation of certain Notch target genes, including HES, HEY, and Snail [10], though the underlying mechanisms are largely unknown.

In this study, we provide evidence for the miR-34a/Notch pathway involvement in hypoxia-induced EMT. Hypoxia may downregulate the expression of miR-34a in renal tubular epithelial cells. Knockdown of miR-34a promoted renal tubular epithelial cells EMT, while the overexpression of miR-34a abolished the occurrence of hypoxia-induced EMT. Moreover, Jagged1 and Notch1 are negatively regulated by miR-34a. Thus, we demonstrate that miR-34a is downregulated by hypoxia and plays a role in kidney epithelial cell EMT by targeting Notch signaling.

## Materials and Methods

### Ethics statement

All subjects filled a questionnaire including their informed consent. The study was carried out according to the Helsinki Declaration and the samples were processed under approval of the written consent statement by Ethical Committee of the Xijing Hospital, Shaanxi province, China.

### Cell culture and experimental conditions

The human proximal tubular epithelial cell line HK-2 was purchased from Bei Na biotechnology Research Institute (Beijing) and preserved in our laboratory [16]. Cells were cultured in DMEM/F12 medium (Invitrogen, Carlsbad, CA) supplemented with 50 units/ml penicillin, 50 mg/ml streptomycin, and 10% fetal calf serum (FCS) (Gibco-BRL Life Technologies, Burlington, Ontario, Canada). The cells were seeded at 1.5×10^6^cells/100 mm diameter dish for 3–4 days at 37°C in10 ml medium in a humidified atmosphere of 5% CO_2_. All experiments were conducted with confluent cultures. For hypoxic culture, cells were placed in a hypoxic (1% O_2_, 5% CO_2_, 37°C) incubator (Precision Scientific, Winchester, VA) for 0, 2, 6, 12, 24, 48 or 72 h. Control cells were incubated for equivalent periods under normoxic conditions (21% O_2_, 5% CO_2_, 37°C).

### Kidney biopsies

Formalin-fixed paraffin-embedded (FFPE) kidney samples were taken from patients with CKD at Xijing hospital from January 2010 to June 2011. Corresponding histopathological and clinical information was collected by chart review. Two to 5 cores (0.6 mm) of tissue were obtained from the FFPE blocks for RNA extraction. FFPE samples of IgA nephropathy (IgAN) (n = 24), diabetic nephropathy (DN) (n = 16) and were identified in the Pathology Department of Xijing Hospital. Nine biopsy samples obtained from living donor biopsies were used as controls.

### miRNA microarray and qRT-PCR

Monolayers of cells (2.5×10^6^) were harvested, and total RNA was extracted with the mirVana miRNA Isolation Kit (Ambion, Inc. Austin, TX) according to the manufacturer's instructions. The isolated miRNAs from the HK-2 cell line were then labeled with Hy3 using the miRCURY™ Array Labeling kit (Exiqon, Vedbaek, Denmark) and hybridized on a miRCURY™ LNA miRNA Array (v10.0, Exiqon) as described [27]. Microarray images were acquired using a Genepix 4000B scanner (Axon Instruments, Union City, CA) and processed and analyzed with Genepix Pro 6.0 software (Axon Instruments) and Excel. The median of four background-corrected replicas for each miRNA capture probe was normalized to the 50th percentile of the positive controls (U6-snRNA-1 and U6-snRNA-2).

Total RNA was extracted from the FFPE tissue cores using the Ambion Recover All Total Nucleic Acid Isolation Kit for FFPE tissues (Applied Biosystems/Ambion, Austin TX, USA) according to the manufacturer's instructions with a modified digestion time (5 hours). RNA was quantitated by the absorbance at 260 nm. qRT-PCR assays were performed as described [28]. For qRT-PCR of mature miRNAs and Notch signal genes, specific primers were designed, and the procedure for reactions is given in table 1. qRT-PCR was conducted according to the manufacturer's instructions (Qiagen; miScript reverse transcription kit, catalog no. 218060; miScript primer assay, catalog no. 218411; and miScript SYBR green PCR kit, catalog no. 218073). Reactions were run on a real-time PCR system (ABI PRISMH 7700, Applied Biosystems). Gene expression was detected with SYBR Green RT-PCR Kit (Qiagen, Inc., Valencia, CA), and relative miRNA and gene expression was determined by normalizing to RUB6 or β-actin using the 2^−ΔΔCT^ method [29].

**Table 1:** qPCR primers and products for notch signal.

gene	Primers(5′to3′)	Size of products
Notch1	ATCCAGAGGCAAACGGAG CACATGGCAACATCTAACCC	89
Notch2	GGACCCTGTCATACCCTCTT CATGCTTACGCTTTCGTTTT	75
Jagged1	AAGGCTTCACGGGAACATAC AGCCGTCACTACAGATGCAC	120
Snail	ACAGAGCATTTGCAGACAGG GTGCTACACAGCAGCCAGAT	147
β-actin	CACTCTTCCAGCCTTCCTTC GGATGTCCACGTCACACTTC	90

### Plasmid constructs and transfection

For luciferase reporter experiments, a 905 bp segment of the 3′-UTR of the Notch1 gene, 495 bp of the 3′UTR of the Notch2 gene, and 366 bp of the 3′UTR of the Jagged1 gene were amplified by PCR from human genomic DNA and inserted into the pMIR-Reporter vector (Ambion, Inc. Austin, TX), using the SpeI and HindIII site immediately downstream from the CMV promoter. The following primer sets were used to generate specific fragments:

Notch1-UTRF5′-CTAGACTAGT TGGCCCCAGGTAGAAACT-3′,

Notch1-UTRR, 5′-CCCAAGCTTGGGACAGGACCAAAGGAC-3′;

Notch2-UTRF, 5′-CTAGACTAGTCAGCTACTGATCCAGCCACT-3′,

Notch2-UTRR, 5′-CCCAAGCTT TCCCTTCCCCACCTCACATA-3′;

Jagged1-UTRF, 5′-CTAGACTAGT ATGACTTAACATAGCCAAAA-3′, Jagged1-UTRR, 5′-CCCAAGCTT AGTATCTTCACGGTCTCAAT-3′. All inserts were confirmed by sequencing.

Plasmid transfections were performed using Lipofectamine 2000 (Invitrogen, Carlsbad, CA, USA) as per the manufacturer's protocol. Fresh medium was added 6 h post-transfection, and RNA and protein were harvested 24 h post-transfection. Transfection of mature microRNAs or inhibitors (Ambion, Inc. Austin, TX) was performed using siPORT NeoFX transfection agent (Ambion, Inc. Austin, TX) according to the manufacturer's protocol. Transfection complexes were added to the cells at a final oligonucleotide concentration of 20 nM, and the medium was replaced 24 h later.

### siRNA for Jagged1 and Notch1

siRNA against Jagged1 were designed, synthesized, and supplied by Ambion. The selected Jagged1 siRNA consisted of the sense strand, 5′-GGUCCUAUACGUUGCUUGUtt-3′, and the antisense strand, 3′-ACAAGCAACGUAUAGGACCtc-5′. The selected Notch1 siRNA contains the sequence; sense 5′-CACCAGUUUGAAUGGUCAAtt-3′; antisence 5′-UUGACCAUUCAAACUGGUGtt-3′. Scrambled siRNAs contained a random sequence content and with no calculated target gene specificity as assessed by BLASTing against all human sequence databases.

HK-2 cells in 25 cm^2^ flasks were transfected with 400 pmol of the annealed RNA primer pair and 10 µl Lipofectamine 2000 (Invitrogen, Carlsbad, CA). Forty-eight hours after transfection, cells were placed into a hypoxic incubator for another 48 h to observe the effect of Jagged1 or Notch1 silencing on the hypoxic cells. The HK-2 cells transfected with Lipofectamine 2000 (mock transfection) or cells transfected with scrambled siRNA were cultured in hypoxic conditions and used as negative controls.

### Immunofluorescence and Immunohistochemistry

For immunofluorescence analysis, HK-2 cells were cultured on sterile glass coverslips in 24-well plates. The slides were fixed with 95% alcohol for 15 min at room temperature. The coverslips were washed with phosphate-buffered saline and permeabilized for 5 min with 0.5% Triton X-100 in phosphatebuffered saline. The cells were then incubated with primary antibodies including anti-Zo-1, anti-E-cadherin, anti-α-SMA, and anti-vimentin (1∶100; Santa Cruz Biotechnology, Santa Cruz, CA) after blocking with 10% normal goat or rabbit serum for 1 h. The slides were incubated with FITC-conjugated goat anti-mouse or anti-rabbit IgG as the secondary antibody at room temperature for 1 h. Slides were examined with a Nikon (Melville, NY) Eclipse TE300 fluorescence microscope, and pictures were taken with a SPOT Diagnostic (Sterling Heights, MI) CCD camera.

For immunohistochemistry, sections approximately 3–5 µm thick of FFPE specimens were made. Slides were deparaffinized in xylene and rehydrated serially with alcohol and water. Endogenous peroxidase activity was blocked with 3% hydrogen peroxide for 30 minutes, followed by heating in a microwave oven for epitope retrieval. The sections were then blocked in 10% normal goat serum and 0.3% Triton X-100 in phosphate-buffered saline (PBS) for 1 h. Expression of snail and E-cadherin were evaluated by immuno-histochemical staining techniques using the avidin-biotin peroxidase complex method using an LSAB2 kit (Dako, Glostrup, Denmark). The primary antibodies used in this study were anti-Snail antibody or E-cadherin (1∶200; Santa Cruz Biotechnology, SantaCruz, CA). Negative control slides using normal mouse immunoglobulin G (Vector Labs, Burlingame, CA) as primary antibody were included in all assays. Immunostaining was evaluated in a blinded manner as described [10]. A 0–3 relative scale was used to grade the amount of Snail immunostaining: 0, <5% staining; 1+, 5–25% staining; 2+, 25–50% immunostaining; 3+, >50% immunostaining. A negative/positive relative scale was used to grade the amount of E-cadherin immunostaining: negative (0), loss or reduced membrane expression of E-cadherin; positive (1), preserved membrane expression of E-cadherin in tubular epithelial cells.

### Protein preparation and Western blot analysis

Harvested cells (2×10^6^) were put into 1.5-mL Eppendorf tubes and homogenized with 400 µL lysis buffer (50 mM Tris-HCl, pH 8.0, 150 mmol/L NaCl, 0.1% SDS, 1% Nonidet P-40, 0.5% sodium deoxycholate 0.02% sodium azide, 100 µg/ml PMSF, 1 µg/ml aprotinin). Cell lysates were centrifuged at 4°C for 5 minutes at 10,000 rpm, and the protein-containing supernatant was placed in fresh tubes and quantified using the Bradford protein assay.

For western blot analysis, total proteins (60 µg) were electrophoresed on 6–10% SDS polyacrylamide gels and then transferred to nitrocellulose membranes (Millipore, Bedford, MA). After blocking with 5% fat-free milk in TBS (20 mmol/L Tris, 0.15 mol/L NaCl (pH 7.0), 0.1% Tween 20), the membranes were incubated with a primary antibody: anti-Notch2, anti-Jagged1, anti-Notch1, and anti-Snail (Cell Signaling Technology) diluted 1∶1000, anti-E-cadherin, anti-Zo-1, anti-α-SMA, and anti-vimentin (1∶200; Santa Cruz Biotechnology, Santa Cruz, CA). After repeated washing, the membranes were incubated with horseradish-peroxidase-conjugated anti-rabbit or anti-mouse secondary antibody (Santa Cruz Biotechnology) diluted 1∶2000. The bands were visualized using the enhanced chemiluminescence system (Amersham Pharmacia Biotech) and exposed to Kodak X-OMAT film (Rochester, New York, USA). Western blot for β-actin was performed as an internal sample loading control by using a mouse monoclonal antibody (1∶5000, Sigma Chemical Co). Autoradiograms were quantified by densitometry (software: Bio Image IQ). Relative protein levels were calculated by normalization to the amount of β-actin protein.

### Target *in vitro* reporter assay

Twenty-four hours before transfection, HK-2 cells were plated at 1.5×10^4^ cells per well in 24-well plates and placed in hypoxic conditions for 48 h. Two hundred nanograms of pMIR-Notch1-3′UTR, pMIR-Notch2-3′UTR, pMIR-Jagged1-3′UTR, or pMIR-REPORT, and 80 ng of pRL-TK (Promega, Madison, WI) were transfected alone or in combination with 40 pmol of miR-34a precursor or precursor control using Lipofectamine 2000 (Invitrogen, Carlsbad, CA) according to the manufacturer's protocol. Luciferase activity was measured 48 h after transfection using the Dual Luciferase Reporter Assay System (Promega, Madison, WI). Firefly luciferase activity was normalized to renilla luciferase activity for each transfected well. Three independent experiments were performed in triplicate.

### Statistical analysis

Each experiment was repeated at least three times. Bands from Western blots were quantified with Quantity One software (Bio-Rad, Hercules, CA). Relative protein and mRNA levels were calculated in comparison to internal β-actin standards. Relative miRNA levels were calculated in comparison to internal RUB6 standards. Numerical data are presented as mean±SD. The difference between means was analyzed with ANOVA. The relationship between the scored Snail or E-cadherin expression and miR-34a expression was assessed by a linear regression correlation test. Differences were considered significant when P<0.05. All statistical analyses were done with the software SPSS12.0 (SPSS Inc. Chicago, USA).

## Results

### MiRNA expression changes under hypoxia in HK-2 cells

**Figure 1:**
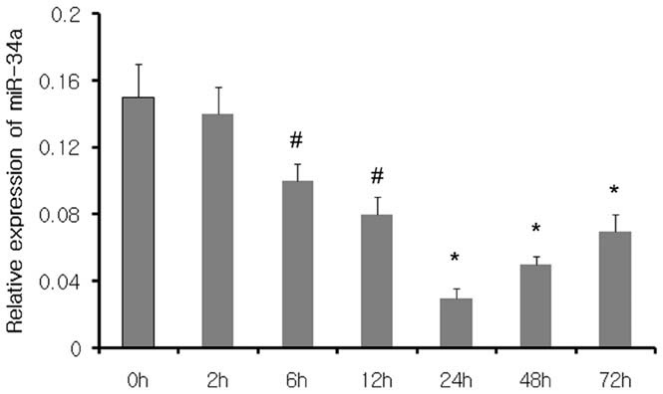
Hypoxia induces downregulation of miR-34a expression in HK2 cells. qRT-PCR analysis of miR-34a expression after 2, 6, 12, 24, 48 and 72 h of hypoxia and the relative amount of miRNA was normalized to U6 snRNA. Triplicate assays were performed for each RNA sample. ^#^p<0.05 and *p<0.01 compared with normoxic controls.

miRNAs were previously demonstrated to be regulated by hypoxia and involved in angiogenesis and tumor progression. Here, we asked whether miRNAs could induce EMT under hypoxia since hypoxia is a key factor in the process of EMT and fibrosis [10]. We performed array-based miRNA profiling of human proximal tubular epithelial cell line under hypoxia for 24 h, 48 h, or normoxia. As shown in table 2, microarray analysis of 533 human miRNAs showed that 17 miRNAs were upregulated and 7 miRNAs were downregulated after 24 h or 48 h of hypoxia in HK-2 cells (ΔΔCt>±1). miR-34a also showed a differential expression ΔΔCt>2 (24 h) and was chosen for further characterization. We then validated the differentially expressed miRNAs by qRT-PCR. Consistent with the microarray findings, miR-34a was shown to be dramatically downregulated after 24 h or 48 h under hypoxia; the decrease starts at 6 h and descended to the lowest level at 24 h and persists through 72 h (Fig. 1).

**Table 2:** Summary of hypoxia-regulated microRNAs in HK2 cells.

microRNA	Symbol	microRNA expression ratio in HK2 cell	
		24/0 h	48/0 h
hsa-miR-30b	up	1.87	2.05
hsa-miR-192	up	2.19	2.51
hsa-miR-193a-3p	up	2.60	1.78
hsa-let-7d*	up	1.83	2.71
hsa-miR-638	up	1.71	2.93
hsa-miR-628-3p	up	1.54	2.35
hsa-miR-553	up	1.58	2.11
hsa-miR-943	up	1.69	2.70
hsa-miR-129-5p	up	2.41	3.24
hsa-miR-9*	up	3.69	4.43
has-miR-194	up	1.59	2.14
hsa-miR-574-5p	up	2.04	2.03
hsa-miR-923	up	2.01	2.28
hsa-miR-376a*	up	1.93	2.26
hsa-miR-493	up	2.15	2.52
hsa-miR-494	up	2.10	2.24
hsa-miR-361-3p	up	1.79	2.17
hsa-miR-34a	down	0.19	0.24
hsa-let-7a	down	0.56	0.43
hsa-miR-601	down	0.40	0.44
hsa-miR-193b*	down	0.39	0.43
hsa-miR-149*	down	0.46	0.29
hsa-miR-25*	down	0.36	0.32
hsa-miR-920	down	0.47	0.44

### miR-34a mediated EMT in tubular epithelial cells under hypoxic conditions

**Figure 2:**
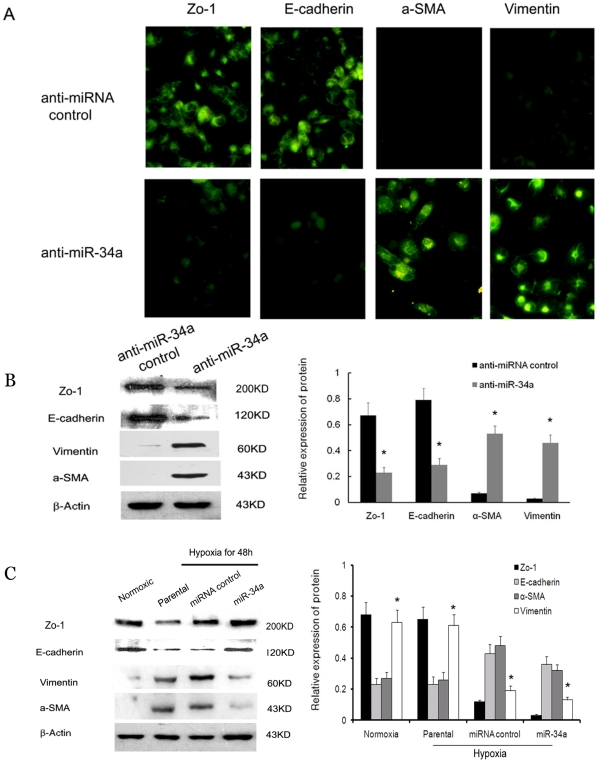
miR-34a mediates hypoxia-induced EMT in HK-2 cells. (A) Immunofluorescence analysis of Zo-1, E-cadherin, α-SMA and vimentin expression in anti-miR-34a and anti-miRNA control transfected HK2 cells. (B) Western blot analysis of Zo-1, E-cadherin, α-SMA and vimentin expression in anti-miR-34a and anti-miRNA control transfected HK2 cells. A representative blot from three independent experiments is shown (left). The histogram shows the average volume density normalized to the loading control, β-actin (right). (C) Western blot analysis of Zo-1, E-cadherin, α-SMA and vimentin expression in parental cells, miR-34a and miRNA control transfected cells after 48 hours under hypoxic conditions. A representative blot from three independent experiments is shown (left). The histogram shows the average volume density normalized to the loading control, β-actin (right). *p<0.01 compared with the parental cells and control cells.

To investigate whether miR-34a has a direct function in hypoxia-induced EMT or is simply differentially modulated in hypoxia-induced HK-2 cells, we used gain-of-function and loss-of-function approaches in HK-2 cells under normoxia or hypoxia. Firstly, we used a miR-34a specific inhibitor to suppress miR-34a expression in HK-2 cells, which highly express endogenous miR-34a under normoxia. Forty-eight hours after transfection, the expression of E-cadherin, ZO-1, α-SMA, and vimentin were detected in cells by immunofluorescence and western blot. As shown in Fig. 2A, E-cadherin, an epithelial marker, was downregulated in cells transfected with the miR-34a inhibitor compared with parental cells and miRNA inhibitor control transfected cells. In contrast, the expression levels of mesenchymal markers, α-SMA and vimentin, were higher in miR-34a inhibitor transfected cells as compared with control cells. These results were confirmed by western blot (Fig. 2B).

Next we asked whether miR-34a is involved in EMT in hypoxia-induced HK-2 cells. The precursor of miR-34a or precursor of the miRNA control was transfected into HK-2 and processed under low oxygen. After another 48 h, the cells were collected, and the expression of E-cadherin, ZO-1, α-SMA and vimentin was detected by western blot. After 48 h of hypoxia, the levels of E-cadherin and ZO-1 protein were significantly reduced as compared with that in normoxic cells, whereas the levels of α-SMA and vimentin protein were upregulated in hypoxic cells (Fig. 2C). Transfection with the miR-34a precursor prevented the increase in E-cadherin and ZO-1 to the level present in normoxic cells. Expression of α-SMA and vimentin were correspondingly reduced in hypoxic cells after transfection with the miR-34a mimic (Fig. 2C).

### Notch signaling is activated by hypoxia and inversely correlated with miR-34a expression

**Figure 3:**
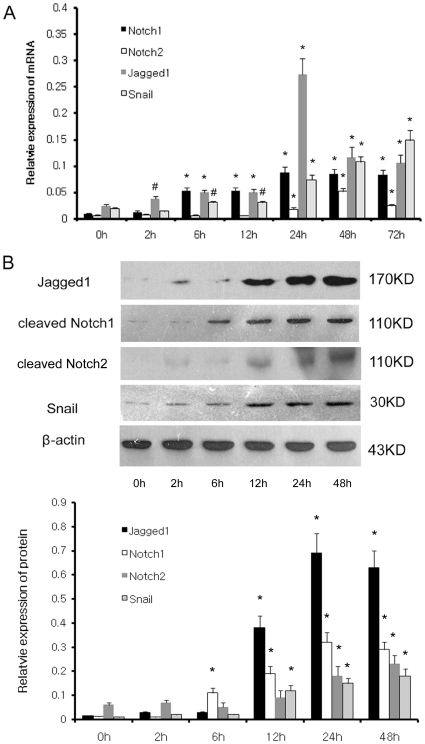
Hypoxia induces Notch signal protein and mRNA expression in HK2 cells. (A) qRT-PCR analysis of Notch1,Notch2, Jagged1, Snail in HK-2 cells after 2 h, 6 h, 12 h, 24 h, 48 h and 72 h of hypoxia and the relative amount of mRNA was normalized to β-actin. (B) Western blot analysis of Notch1, Notch2, Jagged1, Snail in HK-2 cells after 2 h, 6 h, 12 h, 24 h and 48 h of hypoxia. A representative blot from three independent experiments is shown (upper). The histogram shows the average volume density corrected for the loading control, β-actin (lower). ^#^p<0.05 and *p<0.01 compared with normoxic controls.

miR-34a has been demonstrated to function as a tumor suppressor by negatively regulating the expression of many essential oncogenes. Therefore, it is likely that miR-34a could modulate some EMT-related genes and play a role in EMT. By *in silico* analysis, miR-34a is predicted to target several members/genes of notch signaling: Notch1, Notch2 and Jagged1, which were reported to play critical roles in EMT of tumor and endothelial cells. Therefore, we sought to detect the expression of Notch signaling genes under low oxygen conditions and analyze the relationship with miR-34a. Figure 1 showed that the expression of miR-34a in HK2 cells decreased after 6 h under hypoxia, descended to the lowest level at 24 h and remained downregulated for more than 72 h. At the same time, the mRNA levels of notch signaling genes, including Notch1, Notch2, Jagged1, and Snail were dramatically increased *in vitro* under hypoxia (Fig. 3). Notch1, Notch2, and Jagged1 were upregulated at 6, 24, and 2 h after hypoxia and peaked at 24, 48, 24 h, respectively. The hypoxia-induced upregulation remained for more than 72 h. Snail, well known as a Notch signal/CSL target gene, was also shown increased expression after 6 h under hypoxia, peaked at 48 h, and remained upregulated for more than 72 h. Moreover, the results of western blot showed that Notch1 and Jagged1 protein levels were increased after 6 h and 12 h, respectively and both reached a maximum level at 24 h, and remained upregulated for more than 48 h. Apparently, the time-dependent changes of Notch1 and Jagged1 protein levels were not consistent with that of mRNA levels. It was also found that the protein levels, but not mRNA levels, of Notch1 and Jagged1 were inversely correlated with miR-34a under low oxygen from 2 h to 48 h. In our limited analysis, Notch signal protein levels correlate poorly with Notch mRNA levels but are very consistent with miR-34a levels, suggesting that the expression of Notch1 and Jagged1 are significantly influenced in HK-2 at the level of translation, consistent with the known mechanism of miRNAs in invertebrates. The expression of the Notch2 and Snail protein was induced by hypoxia at 6 h and 24 h, respectively, which was in agreement with the mRNA level.

### miR-34a directly targets Jagged1 and Notch1, essential negative regulators of the Notch pathway

**Figure 4:**
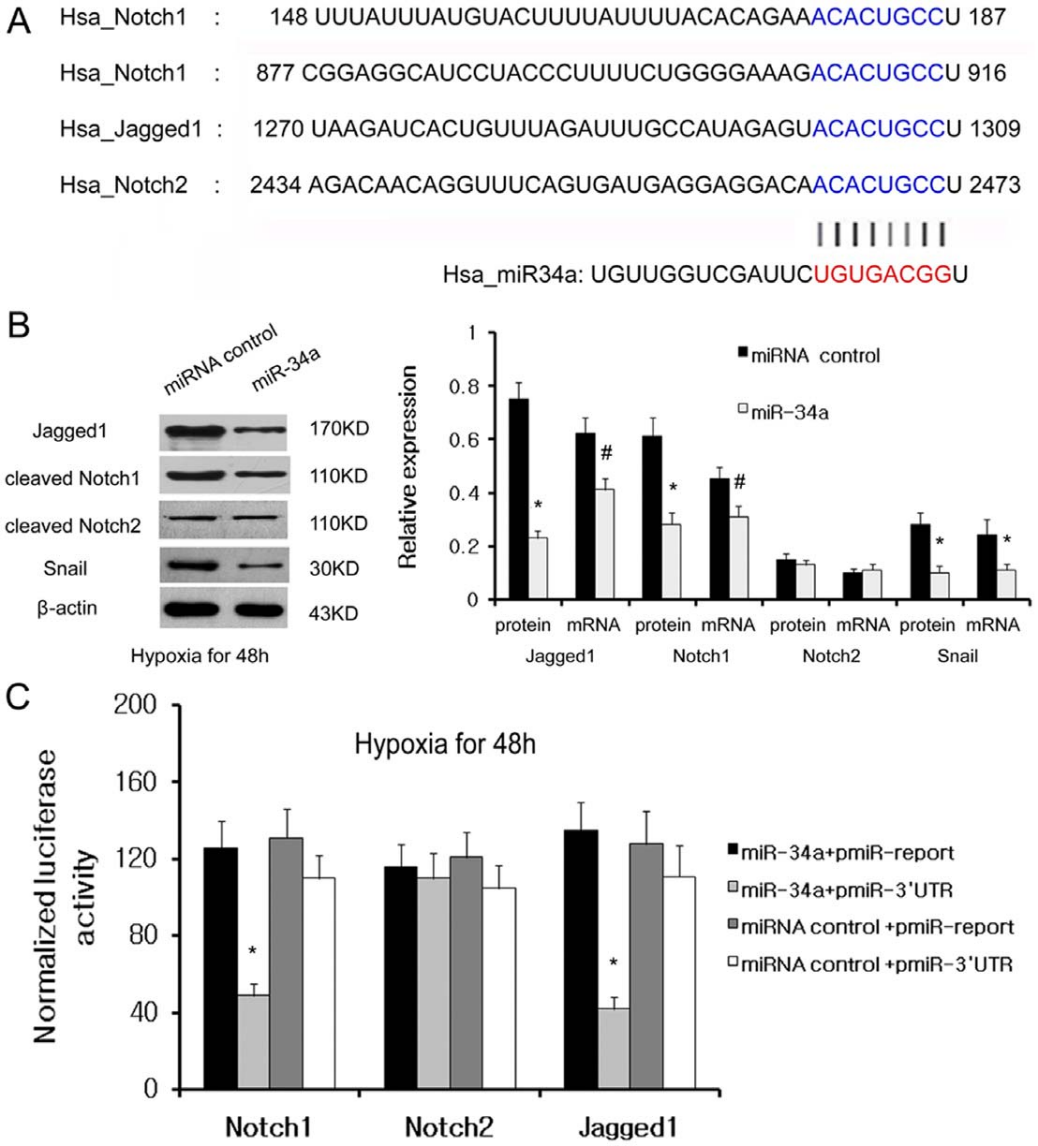
Notch1 and Jagged1 are directly regulated by miR-34a. (A) Putative binding sites of miR-34a in Notch1, Notch2 and Jagge1 3′UTR are shown with color letters. (B) Jagged1, Notch1, Notch2, Snail protein level and mRNA level were respectively detected by Western blotting and qRT-PCR 48 h after transfection with miR-34a precursor or precursor control in HK2 cells under low oxygen. A representative blot from three independent experiments is shown (left). The histogram shows the average volume density normalized to the β-actin (right). (C) Dual luciferase assay was performed in HK2 cells under hypoxia co-transfected with luciferase construct (pMIR-Norch1-3′UTR, pMIR-Norch2-3′UTR, pMIR-Jagged1-3′UTR or pMIR-report control) and miR-34a precursors or precursor control. Firefly luciferase activity was normalized to Renilla luciferase activity for each sample. The results shown represent the mean ± SD from 3 independent experiments. ^#^p<0.05 and *p<0.01 compared with empty vector or miRNA precursor control.

Next, we wanted to validate whether Jagged1 and Notch1 are direct targets of miR-34a in renal epithelial cells. We transfected HK-2 cells with precursors of miR-34a or control miRNAs and determined whether miR-34a negatively regulates Notch signaling in HK-2 cells under low oxygen. The expression of Notch protein and mRNA were detected by western blot and qRT-PCR 48 h after transfection. As shown in Fig. 4, the miR-34a precursor dramatically decreased the expression of Notch1 and Jagged1 protein and mRNA, while there were no effects of miR-34a on Notch2 protein and mRNA levels. Notch1 and Jagged1 proteins were inhibited 54.1% and 69.3% by miR-34a transfection compared with control, respectively, whereas the mRNA of Notch1 and Jagged1 was reduced by 31.1% and 33.9% by miR-34a, respectively. The protein levels of Notch1 and Jagged1 were suppressed more than the mRNA levels by miR-34a transfection, implying that mR-34a regulated the expression of Notch1 and Jagged1 at the post-transcriptional level. We also detected the expression of downstream Notch signaling gene after transfection with miR-34a. The results showed that Snail protein expression was suppressed by miR-34a transfection.

To demonstrate a direct interaction between miR-34a and Notch1, we cloned a 905 bp Notch1 3′-UTR segment, which contains two potential target sites for miR-34a (Fig. 4A), downstream of the pMIR-Report luciferase reporter gene to generate the pMIR-Notch1 vector. This vector was co-transfected into HK-2 cells with miR-34a or a scrambled miRNA negative control under low oxygen. A renilla luciferase vector (pRL-TK) was used to normalize differences in transfection efficiency. Luciferase activity in HK-2 cells co-transfected with pMIR-Notch1 and miR-34a was decreased to 39% when compared with negative control miRNA precursors (P<0.001) (Fig. 4D). Similarly, we cloned a 366 bp Jagged1 3′-UTR segment and a 495 bp Notch2 3′-UTR segment, which contain potential target sites for miR-34a (Fig. 4A), downstream of the pMIR-Report luciferase reporter gene to generate the pMIR-Notch2 and pMIR-Jagged1 vectors. Using the reporter gene assay, we demonstrated that luciferase activity in HK-2 cells co-transfected with pMIR-Jagged1 and miR-34a was decreased to 31% when compared with negative control miRNA precursors, while there was no significant luciferase activity change with pMIR-Notch2. These data indicate that miR-34a directly interacts with the 3′UTR of Notch1 and Jagged1, but not with Notch2. Taken together, the immunoassay and luciferase results provide strong indications that Jagged1, as well as Notch1, are targets of miR-34a in HK-2 cells.

### Jagged1 or Notch1 siRNA prevents miR-34a inhibitor-induced EMT via suppression of Notch signaling

**Figure 5:**
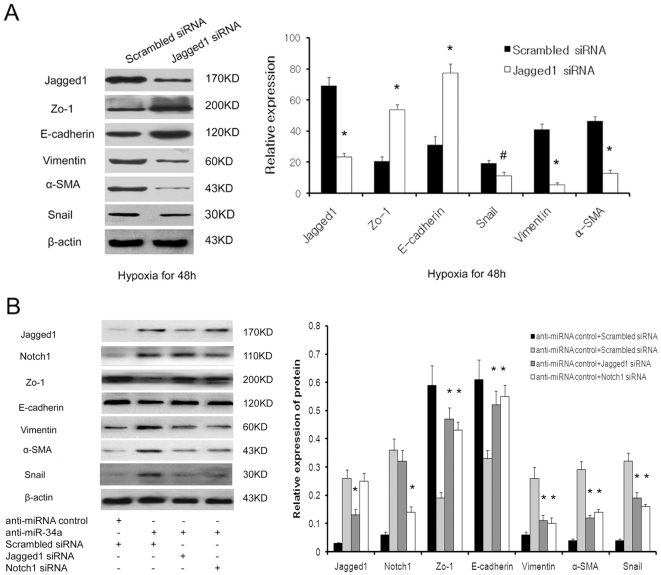
Jagged1 or Notch1 siRNA prevents miR-34a inhibitor-induced EMT via suppression Notch signal. (A) Jagged1 siRNA prevents hypoxia-induced HK2 cells EMT via suppression Notch signal. Western blots analysis of Zo-1, E-cadherin, α-SMA, vimentin and Snail expression in Jagged1-specific or scrambled siRNA transfected cells after 48 hours under hypoxic conditions. A representative blot from three independent experiments is shown (left). The histogram shows the average volume density normalized to the loading control, β-actin (right). (B) Jagged1 or Notch1 siRNA prevents miR-34a inhibitor-induced HK2 cells EMT via suppression Notch signal. Western blots analysis of Zo-1, E-cadherin, α-SMA, vimentin and Snail expression in Jagged1-specific or scrambled siRNA and miR-34a inhibitor cotransfected HK2 cells. A representative blot from three independent experiments is shown (left). The histogram shows the average volume density normalized to the loading control, β-actin (right). ^#^p<0.05 and *p<0.01 compared with the parental cells and control cells.

To assess the effect of Notch signaling on hypoxia-mediated EMT, Jagged1 siRNA and scrambled siRNA was transfected into HK-2, and the cells were moved to a hypoxic incubator (1% O_2_, 5% CO_2_, 37°C) for another 48 h-incubation. As shown in Fig. 5A, the scrambled siRNA transfected HK-2 cells developed an EMT phenotype after 48 h under hypoxia companied by downregulation of Zo-1 and E-cadherin levels and upregulation of vimentin and α-SMA protein levels. Jagged1-specific siRNA could effectively suppress Jagged1 and the downstream molecule Snail and prevent EMT in tubular epithelial cells. HK-2 cells transfected with Jagged1-specific siRNA demonstrated increased levels of Zo-1 and E-cadherin, and reduced α-SMA and vimentin expression compared with negative controls (Fig. 5A).

Next, we sought to investigate whether Notch signaling was required for miR-34a mediated EMT in tubular epithelial cells. HK-2 cells were transiently cotransfected with a miR-34a inhibitor and siRNA targeting Jagged1 or Notch1 or a scrambled siRNA sequence, and the effects on cell EMT were determined as described above. As shown in Fig. 5B, transfection with Jagged1 or Notch1 siRNA prevented the miR-34a inhibitor-mediated increase in Notch signal and Snail. The expression of Zo-1 and E-cadherin were correspondingly increased while vimentin and α-SMA were reduced in miR-34a inhibitor-transfected cells after treatment with Jagged1 or Notch1 siRNA. These results indicate that miR-34a mediates the mesenchymal transition of HK-2 cells through the Notch pathway.

### miR-34a expression was down-regulated in renal tissues of patients with chronic hypoxia kidney diseases

**Figure 6:**
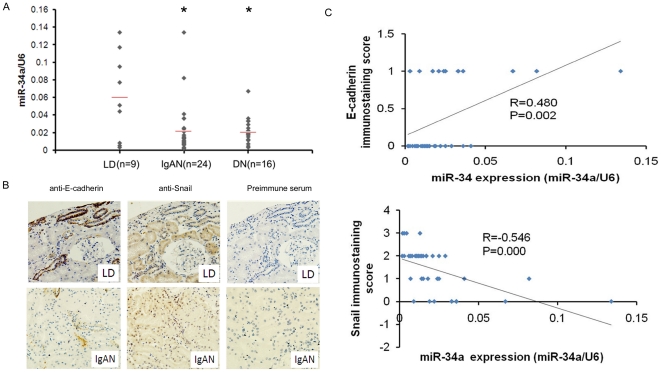
Expression of miR-34a in renal tissue from patients with CKD. (A) Expression analysis of miR-34a in renal tissue from patients with DN, IgA nephropathy (IgAN) by real-time PCR. Shown are relative expression values normalized to U6. Pretransplant biopsies from living donor kidneys (LD) were used as control. *P<0.01. (B) Expression of E-cadherin and Snail in renal tissue from living donor (LD) kidneys and patients with IgA nephropathy (IgAN) by IHC (original magnification, 400×). (C) Scatter plot with fitted values intervals for tubular expression of miR-34a and E-cadherin (upper) or Snail (lower).

Having observed that miR-34a mediates hypoxia-induced EMT in HK-2 cells, we asked if the expression of miR-34a is associated with tubular epithelial cells EMT in human renal tissues of patients with chronic hypoxia kidney diseases. A total of 40 renal specimens from patients with chronic hypoxia kidney diseases (24 cases of IgA nephropathy and 16 cases of diabetic nephropathy) and 9 samples from living donor biopsies were examined for the expression of miR-34a. QRT-PCR analyses revealed reduced miR-34a level in IgA nephropathy and diabetic nephropathy renal tissues compared with normal renal tissues (Fig. 6A, p<0.01 both). We then detected the epithelial marker E-cadherin and mesenchymal marker Snail expression in these samples with immunohistochemistry. As shown in Fig. 6B, strong membranous labeling of E-cadherin and little positive staining of Snail were observed in normal tubular epithelial cells, whereas reduced membranous labeling of E-cadherin and strong nuclei staining of Snail was observed in 65% (26/40) and 55% (22/40) of diseased renal tissues, respectively. These results demonstrated that tubular epithelial cells of diseased human renal tissues are undergoing mesenchymal phenotype. Moreover, by linear analysis, we found that miR-34a expression was correlated with E-cadherin (p = 0.002) and inversely correlated with snail (p = 0.000) (Fig. 6C). These data further support the findings that miR-34a was involved in EMT as observed in HK2 cells.

## Discussion

Tubulointerstitial fibrosis is recognized as a key determinant of progressive chronic kidney disease (CKD). EMT of proximal tubular epithelial cells, driven largely by cytokines (e.g., TGF-β), hypoxia, and other reasons, is integral to this process. Recently, miRNAs, a cluster of newly discovered regulators, were reported to be involved in the development of chronic kidney disease, including hypertensive kidney disease, diabetic nephropathy, and IgA nephropathy [30]–[32]. The expression of miR-200a, miR-200b, miR-141, miR-429, miR-205, and miR-192 were shown to be increased in renal biopsy specimens from patients with hypertensive nephrosclerosis, and were correlated with disease severity, such as levels of proteinuria and eGFR [30]. *In vitro* and animal studies have demonstrated that these miRNAs (miR-200a, miR-200b, and miR-429) repress EMT by inhibiting ZEB1 and ZEB2 [31], [32]. The miR-200a/b precursor could ameliorate renal tubulointerstitial fibrosis through repression of TGF-β2 expression in diabetic nephropathy, suggesting that miR-200 could be a permissible therapeutic target for renal fibrosis [33], [34]. Krupa *et al.* demonstrated that low levels of miR-192 in patients with diabetic nephropathy was correlated with tubulointerstitial fibrosis and impaired eGFR. Overexpression of miR-192 suppressed the expression of E-box repressors ZEB1 and ZEB2, thereby opposing TGF-β-mediated downregulation of E-cadherin [7]. Kato's team further investigated the role of miR-192 in mesangial cells. TGF-β-induced miR-192 was shown to mediate increases in the expression of collagen 1 alpha 2 in murine mesangial cells [35]. In addition, Du and colleagues found that high glucose and TGF-β levels induced a marked reduction of miR-29a expression, which subsequently led to augmented COL4A1 and COL4A2 expression and increased the risk of excess collagen deposition in proximal tubular epithelial cells [36]. Here, we presented for the first time that the downregulation of miR-34a by hypoxia could induce EMT in tubular epithelial cells, adding to the evidence that miR-34a may serve as a biomarker and potential therapeutic target for CKD.

Hypoxia is a common microenvironment for multi-pathophysiologic progress, including tumorigenesis and organ fibrosis [37], [38]. Increasing evidence has shown that miRNA is involved in tumorigenesis, angiogenesis, and organ fibrosis driven by hypoxia. Hua *et al.* demonstrated that miR-15b, miR-16, miR-20a, and miR-20b are sharply downregulated in CNE cells during hypoxia [39]. Studies from Kulshreshtha's group identified a set of hypoxia-regulated miRNAs (HRMs), providing an additional link between a tumor-specific stress factor and gene expression control [40]. When primary fibroblasts were placed under hypoxic stress, only 3 out of 377 miRNA subtypes were downregulated [41]. Our study showed that 17 miRNAs were upregulated and 7 miRNAs were downregulated under hypoxia in HK-2 cells. The disparity may suggest that change in miRNA profile in response to low oxygen is likely to be cell type-specific.

We selected miR-34a, the most differentially expressed miRNA among those that were downregulated, for further experimentation under hypoxic conditions. miR-34a maps to the distal region of chromosome 1p. Genomic deletion or loss of heterozygosity of this chromosomal region has been reported in many types of tumors [42]–[45]. Therefore, loss of heterozygosity of miR-34a, which functions as a tumor suppressor in these tumors, is not surprising. In fact, the importance of miR-34a in cancer was recently well established and shown to have tumor suppressive effects in multiple types of cancers, including hepatocellular carcinoma [46], pancreatic cancer [47], colon cancer [48], and chronic lymphocytic leukemia [49]. More recently, Liu *et al.* [50] showed that miR-34a inhibits prostate cancer stem cells and metastasis by directly repressing CD44, which establishes a strong rationale for developing miR-34a as a novel therapeutic agent against prostate cancer stem cells. Although the direct effects of miR-34a have been studied in a wide range of cancer cells, relatively few studies regarding miR-34a in other cellular functions have been reported. Our data showed that miR-34a is involved in hypoxia-induced tubular epithelial cell EMT. Moreover, we further showed that the expression of miR-34a was reduced in chronic hypoxia renal tissues of IgAN and DN patients compared with normal renal tissues. These results abounded the function of miR-34a in addition to its role as a tumor suppressor.

Next, we tried to investigate the mechanism underlying the involvement of miR-34a in hypoxia-induced EMT. miR-34a has multiple, experimentally validated targets involved in cellular proliferation and apoptosis, such as MYCN, BCL2, SIRT1, SFRP1, CAMTA1, NOTCH1, JAG1, CCND1, CDK6, E2F3, and CD44 [50], [51]. Among these known miR-34a target genes, Notch1 and Jagged1 were shown to promote EMT and renal fibrosis in tubular epithelial cells by activation of the Notch signaling pathway. By *in silico* analysis, Notch1, Notch2, and Jagged1 were identified as putative targets of miR-34a. Both mRNA and protein level of Notch1 and Jagged1 were strongly increased after miR-34a inhibition, while miR-34a mimics reduced Notch1 and Jagged1 mRNA and protein levels to baseline levels. However, the miR-34a inhibitor or mimic had no effect on Notch2 mRNA and protein levels. Luciferase report gene assays further confirmed that Notch1 and Jagged1 were direct targets of miR-34a.

The role of Notch signaling in renal diseases has been well established. The expression of Jagged-1 was found to be upregulated during renal fibrotic disease in a TGF-β-dependent manner [52]. Zavadil's *in vitro* data demonstrated the activation of Jagged1/Notch and Hey1/Notch signaling in TGF-β induced EMT [53]. Recently, a well-performed study by Niranjan and colleagues showed that the Notch pathway in podocytes is important during the development of glomerular disease [54]. A comprehensive study encompassing all Notch ligands and receptors in chronic kidney diseases showed that cleaved Notch1, Notch2, and Jagged1 expressionin podocytes in proteinuric nephropathies was correlated with the amount of proteinuria, and the expression of cleaved Notch1 in the tubulointerstitium was correlated with the severity of tubulointerstitial fibrosis [55]. More recently, Bielesz *et al.* found that expression of Notch in renal tubular epithelial cells was necessary and sufficient for tubulointerstitial fibrosis development, and genetic deletion of the Notch pathway in tubular epithelial cells reduced renal fibrosis [26]. These results indicated that activation of the Notch1/Jagged1 pathway is a common mechanism in the process of tubular cell EMT and renal fibrosis, in addition to the development of glomerular disease.

Notch1 activity influenced by hypoxia may be tissue-specific [56], [57]. In hypoxic lung cancer cells, hypoxia leads to increased expression of Notch1 through a HIF-1α–dependent induction of Notch1 mRNA. Similarly, in melanoma development, Notch1 is transcriptionally regulated [57], [58]. However, in renal cell carcinomas, activation of the Notch pathway is independent of HIF-1α and HIF-2α [59], while in stem and precursor cells, hypoxia regulates Notch1 activity post-translationally via HIF-1α [60]. Notch1 activity has also been reported to be regulated by a factor inhibiting HIF-1α (FIH), and Notch1 itself potentiates the cellular hypoxic response by increasing the recruitment of HIF-1α to the HRE sequences of canonical HIF-1 target genes [61]. In this study, our experiment data showed that hypoxia results in an increased expression of Notch1 mRNA and protein in a HIF-1α–dependent manner. The increase in protein level was much greater than the mRNA level, which demonstrates that Notch1 is regulated transcriptionally and post-transcriptionally. The diverse findings of these studies underline an intricate mechanism of regulation of the Notch complex by its microenvironment through HIF-1α, which may be tissue-specific. There is little evidence showing that hypoxia can induce Jagged1 expression. Hiyama [62] and co-workers recently reported that hypoxia can induce Jagged1 mRNA expression in the annulus fibrosus of rat disc tissue, though the possible mechanism was not explored. Our experiment data demonstrated that Jagged1 was regulated post-transcriptionally by miR-34a under hypoxia.

In summary, the results reported here present the first evidence that miRNAs are involved in the development of hypoxia-induced EMT in tubular epithelial cells. Hypoxia-mediated downregulation of miR-34a could promote EMT in tubular epithelial cells by modulating the Notch signaling pathway. Our limited data provide a novel insight into the mechanisms of hypoxia-induced EMT and a strategy to circumvent this formidable problem.
